# *PORA1/2*-dependent chlorophyll biosynthesis coordinates with carotenoid accumulation to drive petal color patterning in *Liriodendron*

**DOI:** 10.48130/forres-0025-0013

**Published:** 2025-07-14

**Authors:** Lingfeng Hu, Yongwei Zhu, Long Yu, Lu Lu, Yingxuan Ma, Renhua Zheng, Jinfang Zhang, Longying Pan, Jinhui Chen, Zhaodong Hao, Jisen Shi

**Affiliations:** 1 State Key Laboratory of Tree Genetics and Breeding, Co-Innovation Center for Sustainable Forestry in the Southern China, Nanjing Forestry University, Nanjing 210037, China; 2 Fujian Academy of Forestry, Fuzhou 350012, China; 3 Fujian Jinsen Forestry Co. Ltd, Sanming 353399, China

**Keywords:** *Liriodendron*, Petal coloration, Chlorophyll biosynthesis, *PORA* gene, Carotenoid metabolism

## Abstract

*Liriodendron* is a highly valued ornamental genus renowned for its distinctive tulip-shaped flowers. Despite its horticultural importance, the molecular mechanisms underlying interspecific variation in petal coloration, namely green petals in *Liriodendron chinense* (Hemsl.) Sargent, an orange-yellow basal band in *Liriodendron tulipifera* Linn., and an extended orange-yellow band in their hybrid, remain poorly understood. By integrating morphological, transcriptomic, and metabolomic analyses, we found that orange-yellow pigmentation during petal development is closely associated with chlorophyll degradation and carotenoid biosynthesis. The expression of chlorophyll synthesis genes *PORA1* and *PORA2* showed a strong positive correlation with chlorophyll content, and their downregulation led to disrupted chloroplast structure and reduced chlorophyll levels. Concurrently, carotenoid biosynthesis genes *CRTISO* and *LCYE* were markedly upregulated during the formation of the colored petal band. These results highlight the synergistic roles of chlorophyll and carotenoid metabolism in determining petal color patterning in *Liriodendron*, providing a genetic basis for the targeted breeding of ornamental traits.

## Introduction

Flower color is a key determinant of ornamental value, breeding direction, and pollinator attraction^[1,2]^. Petals possess upper and lower epidermis layers, along with palisade and spongy mesophyll tissues, where pigments are spatially localized in specific cell types^[3]^. Anthocyanins, carotenoids, and betalains are the three major classes of pigments, each localized to specific cellular compartments: anthocyanins and betalains in vacuoles, and carotenoids in plastids^[4,5]^. Among these, carotenoid accumulation in chromoplasts produces orange-yellow or red pigmentation in plant organs, whereas carotenoids synthesized in chloroplasts primarily serve photoprotective functions without contributing to visible coloration^[6]^. This disctinction is particularly evident during fruit ripening in species such as tomatoes^[7]^ and citrus^[8]^, where chloroplasts progressively differentiate into chromoplasts, allowing carotenoid accumulation and driving the color transition from green to ripe hues. A comparable transition occurs in the medial stripes of *Liriodendron tulipifera* petals, which initially appear green due to active chlorophyll biosynthesis and chloroplast presence. As development proceeds, chlorophyll degradation leads to a transition toward orange-yellow pigmentation. In contrast, *Liriodendron chinense* petals deepen in green coloration during development^[9]^. These divergent developmental trajectories suggest that species-specific regulation of chlorophyll metabolism underlies petal color divergence in the *Liriodendron* genus.

Recent studies have highlighted the importance of chlorophyll homeostasis in petal coloration. In *Paeonia suffruticosa*, persistent green pigmentation of petals is maintained by continued expression of chlorophyll retention-related genes *CHLOROPHYLLASE 1* (*CLH1*) and light-harvesting complex genes *LHCB1/LHCB5*^[10]^. In contrast, the *Arabidopsis* transcription factor TEOSINTE BRANCHED 1/CYCLOIDEA/PCF4 (TCP4) negatively regulates chlorophyll biosynthesis by direct binding to the promoters of protochlorophyllide oxidoreductase gene *PORB* and divinyl reductase gene *DVR*, thereby inhibiting the greening process^[11]^. Similarly, in *Chrysanthemum morifolium*, a R1-type MYB transcription factor REVEILLE2 (RVE2) repressed the expression of three chlorophyll biosynthetic genes, thereby inhibiting chlorophyll biosynthesis in chrysanthemum ﬂowers^[12]^. Ontogenetic petal color transitions driven by chlorophyll degradation represent a conserved developmental mechanism across diverse angiosperm taxa, including *Capsicum annuum*, *Kalanchoe blossfeldiana*, and *Orychophragmus violaceus*, where initial green pigmentation, conferred by chlorophyll, is progressively removed from distal petal regions through programmed catabolism^[11]^. This targeted chlorophyll clearance facilitates the subsequent accumulation of anthocyanins, carotenoids, or other secondary pigments, resulting in species-specific and developmentally timed color patterns. Collectively, these findings underscore the evolutionary conservation of chlorophyll turnover and plastid transformation as fundamental prerequisites for floral pigment diversification and color patterning across angiosperms.

Plastids are multifunctional organelles involved in photosynthesis and the biosynthesis and storage of carbohydrates, fatty acids, and terpenes^[13]^. Based on pigment content and metabolic activity, plastids are broadly classified into chloroplasts, chromoplasts, leucoplasts, and other specialized forms^[14]^. Plastid interconversion occurs in response to developmental cues or environmental stimuli. Chromoplasts, which serve as carotenoid-rich storage sites, originate via two principal developmental routes: direct differentiation from proplastids or amyloplasts (e.g., in *Carica papaya*^[15]^ and *Citrus sinensis*^[16]^, or redifferentiation from mature chloroplasts (e.g., in *Solanum lycopersicum*^[17]^ and *Mangifera indica*^[18]^).

Carotenoid biosynthesis is orchestrated by both structural genes and complex regulatory networks. Environmental cues such as light and temperature modulate carotenoid accumulation through transcriptional and epigenetic mechanisms. In *A. thaliana* leaves, the phytochrome-interacting factor PIF1 down-regulates carotenoid accumulation by directly repressing the expression of *PSY* (encoding phytoene synthase)^[19]^. Additionally, the histone methyltransferase Set Domain Group 8 (SDG8) promotes carotenoid biosynthesis by targeting *CRTISO* (encoding carotenoid isomerase)^[20]^. In fruit crops, transcriptional regulation of carotenoid biosynthesis is comparatively well characterized. In tomato, multiple MADS-box transcription factors regulate key carotenoid biosynthetic genes^[21]^. The tomato APETALA2a (AP2a) transcription factor activates the expression of *PSY1*, *CRTISO*, *BCH* (encoding zeaxanthin epoxidase), and *PDS* (encoding phytoene desaturase), while simultaneously repressing *ZEP1* (encoding zeaxanthin epoxidase), thereby fine-tuning the carotenoid profile^[22]^. In apple (*Malus domestica*), AP2-34 enhances the expression of *PSY*, leading to increased accumulation of phytoene and *β*-carotene^[23]^. Similarly, in citrus, overexpression of *MADS6* activates *PSY*, *PDS*, *CRTISO*, *LCYB* (encoding lycopene *β*-cyclase), and *BCH*, while repressing *LCYE* (encoding lycopene *ε*-cyclase), thereby redirecting flux within the carotenoid biosynthetic pathway^[24]^.

The genus *Liriodendron* (Magnoliaceae) represents a tertiary relict lineage with only two extant sister species: *Liriodendron chinense* and *Liriodendron tulipifera*, which exhibit a classic East Asia-North America disjunct distribution^[25]^. Their interspecific hybrid, *Liriodendron × sino-americanum*, displays pronounced heterosis, enhanced floral coloration, and extended blooming duration, making it both a valued ornamental species and a fast-growing timber species in China. Previous studies attributed the orange-yellow coloration of *L.* *tulipifera* petals to carotenoid accumulation^[9]^. In this study, we integrated morphological, transcriptomic, and metabolomic analyses across *Liriodendron* taxa to investigate the molecular basis of petal pigmentation. We demonstrate that spatial silencing of *PORA1/2* inhibits chlorophyll biosynthesis, contributing to petal color pattern divergence between the two *Liriodendron* species. Subsequent upregulation of carotenoid biosynthetic genes, particularly *LCYE* and *CRTISO*, facilitates localized carotenoid accumulation, resulting in species-specific petal color patterning. Our findings provide a mechanistic framework for understanding the coordinated regulation of chlorophyll degradation and carotenoid biosynthesis in floral tissues and offer valuable insights for molecular breeding and ornamental improvement in *Liriodendron*.

## Materials and methods

### Plant materials and phenotypic data collection

Fully opened *Liriodendron* petals were selected as experimental materials in this study, including the two species *L. chinense* (Longwangshan, China), *L. tulipifera* (Tennessee, USA), and *L. sino-americanum* (new variety 'JinZhan 1/2', characterized by large, stable flowers with petals almost entirely covered by bright orange-yellow pigment except at the base) grown in Longshan (Anji, China). Petal and pigmented region dimensions (length and width) were measured on six flowers per experimental group. For each flower, three petals (from both inner and outer whorls) were measured, with three replicates per petal; mean values were calculated for further analysis. For transcriptomic and metabolomic profiling, petal samples were collected from individual trees of each genotype (*L. chinense* (Lc), *L. tulipifera* (Lt), and 'JinZhan 1/2' (JZ1/2)) with three biological replicates per genotype. All tissues were snap-frozen in liquid nitrogen within 2 min of excision and stored at −80 °C until RNA isolation and metabolite quantification.

According to the color distribution on the petals of *L. tulipifera*, each petal was divided into three regions, i.e., upper, middle, and lower. To quantitatively assess petal color variation, the colorimeter (CM2300d, KONICA MINOLTA) was used to measure the color values of corresponding regions on both the outer and inner petals of selected samples. Six flowers were analyzed in each experimental group, with three technical replicates performed for each petal region (upper, middle, lower). The CIE L*, a*, b* system is a three-dimensional color space defined by the International Commission on illumination (CIE), with dimension L* for lightness, and a* and b* for the color-opponent dimensions of redness–greenness and blueness–yellowness, respectively. The chroma value (C*), which reflects the colorfulness of a color, of *Liriodendron* petals was calculated using the following formula:

$ \mathit{C}^{{*}} \mathrm{=[(} \mathit{a}^{{*}} \mathrm{)}^{ \mathrm{2}} \mathrm{+(} \mathit{b}^{ {*}} \mathrm{)}^{ \mathrm{2}} \mathrm{]}^{ \mathrm{1/2}} $

### Freehand section and chloroplast autofluorescence statistics

The samples were sectioned freehand by the double-blade method. The procedure was as follows: 1) Fresh petal tissues were cut into 1−2 cm segments and placed in distilled water; 2) Prepared segments were transferred to a smooth glass Petri dish, sectioned perpendicularly to the tissue plane, and then placed in distilled water for subsequent use; 3) Samples were examined and photographed using an inverted microscope (Axio Vert. A1; Zeiss) and a confocal laser scanning microscope (LSM 800 system; Zeiss). The relative intensity of chloroplast autofluorescence was quantified using Image J software (version 1.8.0).

### Petal transcriptome sequencing and functional profiling

Transcriptome sequencing of petals was performed using the Illumina HiSeq platform, generating 150 bp paired-end reads. After quality control, clean reads were obtained and used for *de novo* transcriptome assembly with Trinity (version 2.4.0)^[26]^. The longest Cluster sequences were obtained by hierarchical clustering of Corset. Functional annotation of unigenes was conducted using seven databases: NCBI non-redundant protein sequences (Nr), NCBI non-redundant protein sequences (Nt), Pfam, Eukaryotic ortholog groups (KOG), Swiss-Prot, Kyoto Encyclopedia of Genes and Genomes (KEGG), and Gene Ontology (GO). Clean reads from each sample were mapped to the reference transcriptome generated by Trinity. Gene-level read counts were obtained using RSEM^[27]^, based on alignment results from Bowtie, and subsequently converted to TPM values to quantify expression levels. Differential expression analysis was conducted using the R package DESeq2^[28]^, with significance thresholds set at adjusted *p* < 0.05 and |log_2_FoldChange| > 1. Clustering of differentially expressed genes was performed using the Short Time-series Expression Miner (STEM) software (version 1.3.13). KEGG pathway enrichment analysis was conducted using the Bioconductor package clusterProfiler (v4.4.4)^[29]^. The sequence data have been deposited in the NCBI SRA under accession number SRX19229753−SRX19229764.

### Determination of chlorophyll and carotenoid content

The determination of carotenoid content was conducted following the method described by Hao et al.^[9]^. Chlorophyll content in petal samples was measured using spectrophotometry. Fresh petal tissue (1 g) was ground and extracted with 80% acetone, then filtered, and the final volume was adjusted to 50 mL. The absorbance of the extract was determined at 663 and 645 nm. Chlorophyll content was calculated using the following formulas:

$ {\mathrm{Chlorophyll}}\; {a}\; ({\text {µ}}{\rm g}\cdot {\rm g}^{-1})=(12.214\times {\rm A}_{663}-2.81\times {\rm A}_{645})\times 50 $

$ {\mathrm{Chlorophyll}} \;\mathit{b} \;{({\text µ}{\mathrm g}\cdot {\mathrm g}^{-1})=(20.13\times {\mathrm A}}_{ \mathrm{663}}- \mathrm{5.03\times A}_{ \mathrm{645}} \mathrm{)\times 50} $

### Gene cloning, sequence analysis, and vector construction

*LCYE* genes were amplified from Lc, Lt, and JZ1/2 cDNAs using the cloning primers CL_LCYE-F/R1 and CL_LCYE-F/R2 (Supplementary Table S1) with Phanta Max Super-Fidelity DNA Polymerase (Vazyme, Nanjing, China). PCR products were cloned into the pMD™ 19-T vector (Tsingke, Nanjing, China) and transformed into Trelief® 5*α* competent cells (Tsingke, Nanjing, China). Positive clones were selected by colony PCR, cultured overnight on LB medium containing ampicillin, and verified by Sanger sequencing. A phylogenetic tree of the *LCY* gene family was constructed using IQ-TREE2^[30]^, encompassing representatives form two monocots (*Oryza sativa*, *Zea mays*), six eudicots (*A. thaliana*, *Populus trichocarpa*, *Vitis vinifera*, *Malus pumila*, *Solanum lycopersicum*, *Glycine max*), and five basal angiosperms (*Amborella trichopoda*, *Nymphaea colorata*, *L. chinense*, *L. tulipifera*, *L. sino-americanum*).

For *PORA1/2* VIGS constructs, ~300 bp fragments from the conserved 3' regions were amplified for homologous recombination. These fragments were inserted in reverse orientation into the TRV2 vector using *Xba*I and *Sac*I restriction sites. Recombinant plasmids (TRV2-target gene) were constructed using the ClonExpress II One Step Cloning Kit (Vazyme) and transformed into DH5*α* competent cells (Trelief® 5*α*, Tsingke). Positive clones were confirmed by sequencing, and validated plasmids were subsequently introduced into *Agrobacterium tumefaciens* EHA105 competent cells (WEIDI).

### *Agrobacterium*-mediated VIGS in *L. chinense* petals

*Agrobacterium* strains carrying TRV1, TRV2, or TRV2-*LcPORA1/2* were each inoculated (1 mL) into 50 mL LB liquid medium supplemented with 50 mg·L^−1^ kanamycin and 50 mg·L^−1^ acetosyringone. Cultures were incubated at 28 °C and 200 rpm for 6−8 h, then centrifuged at 5,000 rpm and 4 °C for 10 min. The resulting bacterial pellets were resuspended in infiltration buffer (10 mmol·L^−1^ MgCl_2_, 10 mmol·L^−1^ MES, 50 mmol·L^−1^ AS, pH 5.7) and adjusted to an OD_600_ of 0.8.

TRV2 and TRV2-*LcPORA1/2* suspensions were each mixed with TRV1 at a 1:1 volume ratio. After a 2 h dark incubation at room temperature, the mixtures were used to infect *L. chinense* petals. Healthy flowers were surface-sterilized, and isolated petals were immersed in the bacterial suspensions. Infection was carried out at 28 °C with shaking at 200 rpm for 10 min. Petals were then rinsed 2−3 times with sterile water, surface-dried, and transferred to 1/2 MS solid medium for 24 h of dark culture at room temperature. Finally, the materials were cultured in a growth chamber (20−25 °C) under standard conditions. Phenotypes were assessed after 5−7 d.

### Transmission electron microscopy

Petal tissues were dissected into 5 mm × 5 mm segments and fixed in 2.5% (v/v) glutaraldehyde at 4 °C for 12 h under light-protected conditions. Samples were then washed four times (10 min per wash) with 0.1 M phosphate-buffered saline (PBS, pH 7.4). Secondary fixation was performed in 1% (v/v) osmium tetroxide for 2 h at room temperature, followed by three rinses in PBS. Gradual dehydration was carried out through an ethanol series (30%, 50%, 70%, 85%, 95%, and 3 × 100%; 15 min per step). Dehydrated samples were infiltrated and embedded in LR White resin (Sigma-Aldrich, UK). Ultrathin sections (50−70 nm) were prepared using a Power TomeXL ultramicrotome (RMC Boeckeler, USA), post-stained with 2% (w/v) uranyl acetate for 15 min and 2.5% (w/v) lead citrate for 5 min, and subsequently examined under a Hitachi JEM-1400 transmission electron microscope (Hitachi High-Technologies, Japan) at an accelerating voltage of 80 kV.

### qRT-PCR

The presence of multiple *LCYE* transcripts in petals of different species complicates expression analysis. However, sequence alignment revealed high conservation among the unigenes (Supplementary Fig. S1 & S2). Therefore, primers were designed to target conserved regions of *LCYE*. Petal central regions from Lc, Lt, and JZ1 at various developmental stages were used to analyze the dynamic expression patterns of key genes involved in carotenoid and chlorophyll biosynthesis (Supplementary Table S1). Total RNA was extracted using the KK Fast Plant Total RNA Kit (Zoman, Beijing, China). Quantitative real-time PCR (qRT-PCR) was performed using the SYBR® Green Premix Pro Taq HS qPCR Kit (Vazyme, Nanjing, China) on a LightCycler 480 II system (Roche). Gene expression levels were normalized to *Actin*, based on three biological replicates with technical triplicates each. Relative expression was calculated using the 2^−ΔΔCᴛ^ method^[31]^.

## Results

### Differences in the *Liriodendron* petal phenotype

**Figure 1:**
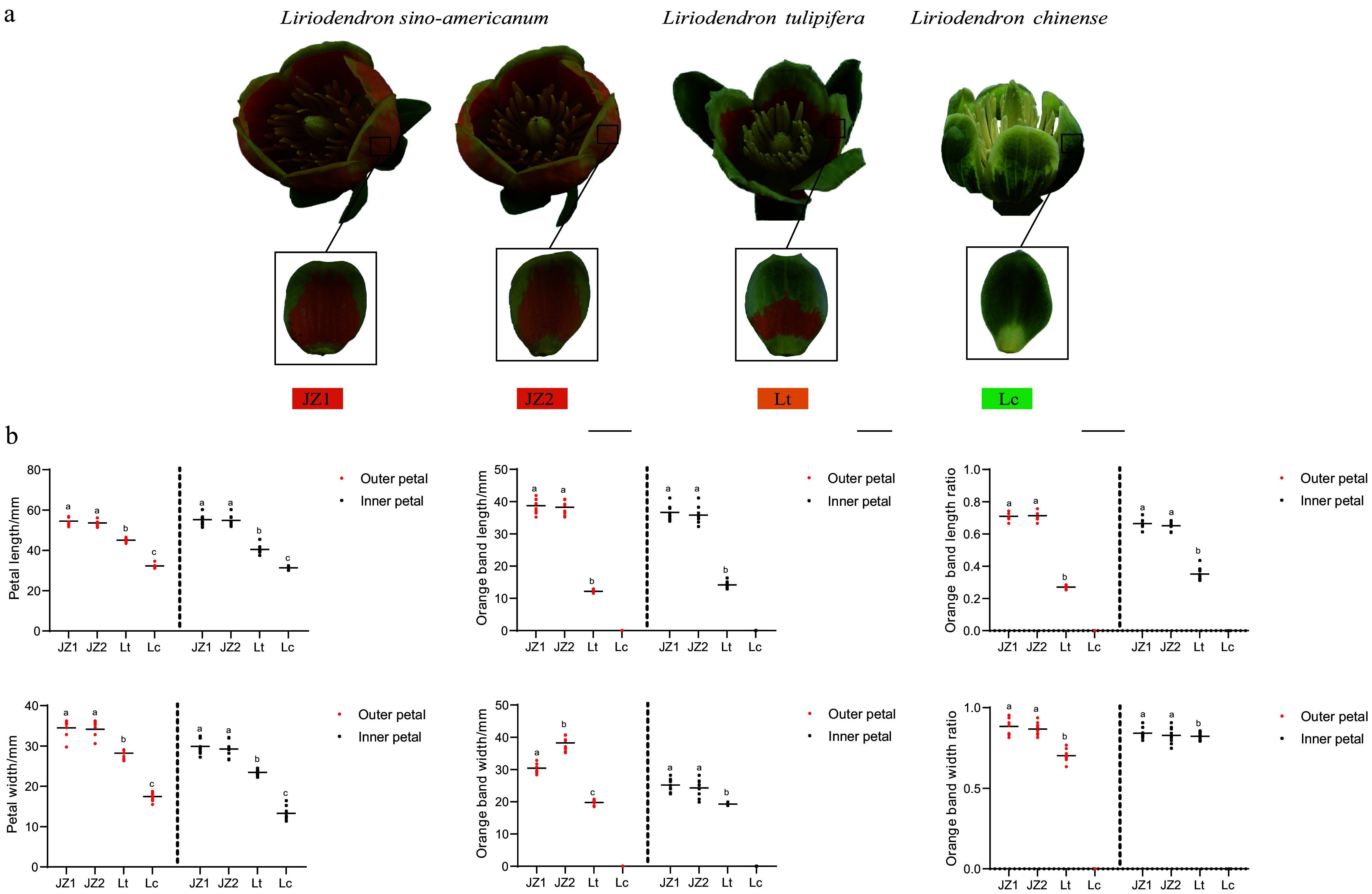
Phenotypic characteristics of *Liriodendron* petals. (a) Representative petal phenotypes of *Liriodendron* samples. Scale bar = 1 cm. (b) Comparative analysis of phenotypic differences between inner and outer petals*.* Data were analyzed by two-way ANOVA followed by Tukey's post-hoc test. Results are presented as mean ± SD. Different letters indicate statistically significant difference (*p* < 0.05).

The variation in petal color of *Liriodendron* contributes to its unique ornamental value, with dark green and orange-yellow petals being the most common. To characterize petal morphology and pigmentation patterns, petals at the blooming stage were dissected from different genotypes, i.e., *L. chinense* (Lc), *L. tulipifera* (Lt), and *L. sino-americanum* (JZ1 and JZ2). Morphometric analysis revealed that both the petal area and the dimensions (length and width) of the pigmented bands in the JZ1 and JZ2 were significantly greater than those in Lt and Lc (Fig. 1a & b). The length-to-width ratio of the orange pigmented region on the outer petals was also significantly greater in JZ1 and JZ2 compared to Lt and Lc (Fig. 1a & b). These results indicate that the flowers of JZ1 and JZ2 are larger and display stable coloration, with petals almost entirely covered by vivid orange-yellow pigments (except at the base), thus conferring a relatively high ornamental value. Further analysis showed that inner petals were narrower than outer petals across *Liriodendron* species. However, the proportion of the orange pigmented area was significantly greater in inner petals than in outer petals in Lt (Supplementary Fig. S3). Additionally, although JZ1 and JZ2 exhibited similar morphological traits and pigmentation patterns, the outer petals of JZ2 were significantly wider than those of JZ1 (Fig. 1b; Supplementary Fig. S3), suggesting that JZ2 may have superior visual appeal.

### Different types of plastids contribute to color variation in *Liriodendron* petals

**Figure 2:**
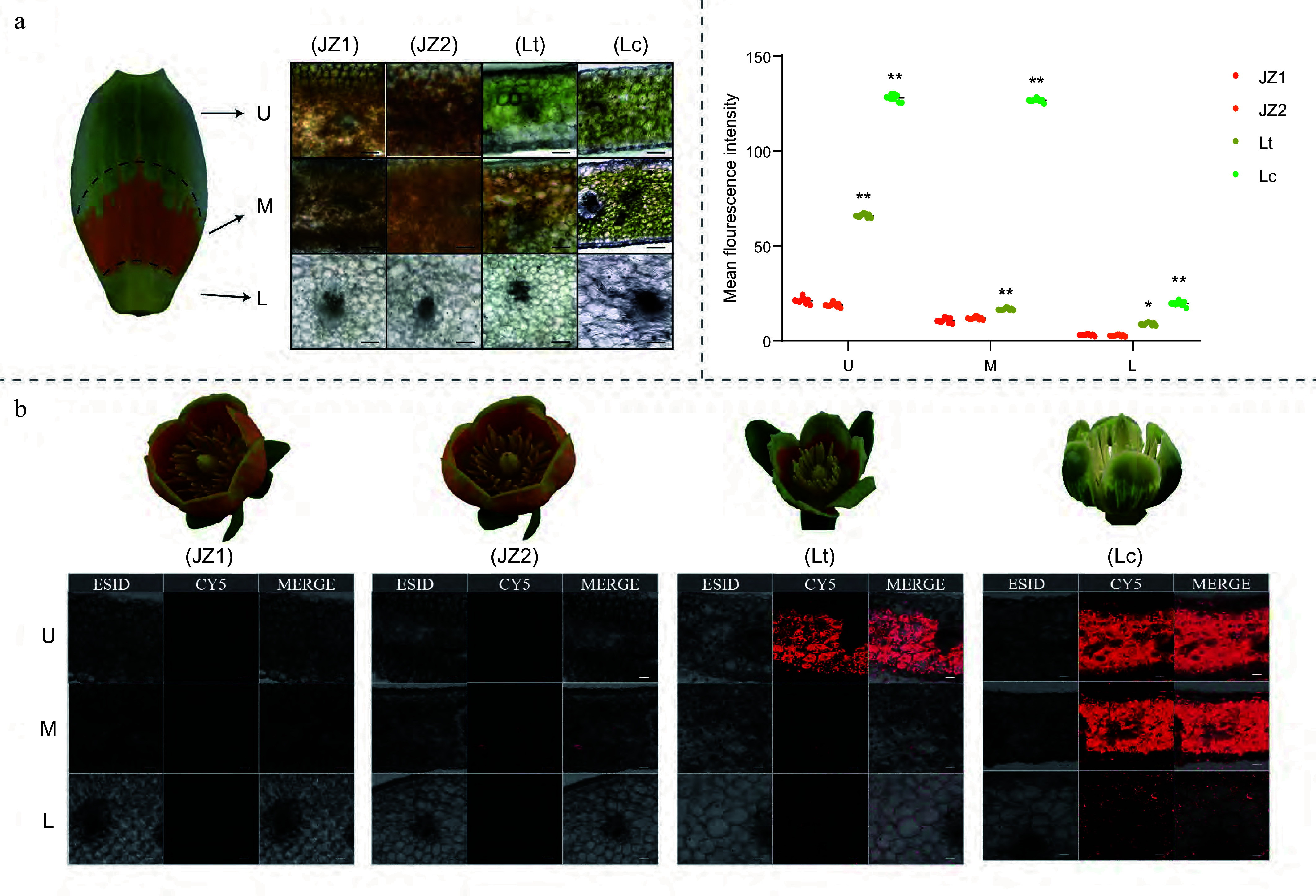
Microstructure of *Liriodendron* petals. (a) Cross sections of different regions of *Liriodendron* petals. U: upper region of the petal. M: middle region of the petal. L: lower region of the petal. Scale bar = 50 μm. (b) Chlorophyll distribution in different petal regions during *Liriodendron* flower development. Scale bar = 50 μm. (c) Chlorophyll fluorescence intensity in different petal regions. Data were analyzed using two-way ANOVA followed by Tukey's post-hoc test. Results are presented as mean ± SD. * and ** indicate statistically significant differences at the *p* < 0.05 and *p* < 0.01 levels, respectively.

To further investigate the pigmentation patterns of *Liriodendron* petals, each petal was divided into three regions based on visible coloration: U (upper), M (middle), and L (lower) (Fig. 2a). The spatial distribution of floral color parameters was quantified using the CIE color system. The lightness (L*) values in the U and M regions of Lc were lower than those of Lt and JZ1/2 (Supplementary Fig. S4). Chroma (C*) was significantly greater in the U and M regions of JZ1/2, and in the M region of Lt, compared to Lc, with the M region of JZ2 exhibiting the highest saturation (Supplementary Fig. S4). In contrast, L* and C* values in the L region remained relatively consistent across all *Liriodendron* samples. The hue a* represents the red-green axis (positive = red, negative = green). In the U and M regions, the general trend was JZ1/2 > Lt > Lc. Specifically, these regions in JZ1/2 and the M region in Lt appeared light red (a*: 27.06~37.01), while those in the U and M regions of Lc and the U and L regions of Lt appeared light green (a*: −1.41 ~ −11.34). The L regions of JZ1/2 and Lc exhibited pale yellow (a*: 5.42~8.11) (Supplementary Fig. S4). The hue b* represents the yellow-blue axis (positive = yellow, negative = blue). The M and U regions of JZ1/2 and Lc, as well as the M region of Lt, showed strong yellow hues (b*: 59.66~77.98). The U region of Lt and the L regions across all samples were characterized by light yellow (b*: 30.62~50.69) (Supplementary Fig. S4). Notably, b* values in the U and M regions of JZ1/2 were higher than those in the corresponding regions of Lc and Lt (Supplementary Fig. S4). These findings illustrate the distinct spatial distribution of petal coloration in *Liriodendron* species based on quantification using the CIE color system.

The relationship between the differently colored regions of *Liriodendron* petals and their internal tissue structures was investigated using anatomical methods. In the L regions of the petals, tissues appeared relatively transparent with no obvious pigment accumulation across *Liriodendron* species (Fig. 2a; Supplementary Fig. S5). In contrast, the U and M regions exhibited species-specific coloration patterns. Abundant chloroplasts were observed in the mesophyll cells of Lc petals, consistent with their characteristic green coloration. Similarly, dense chloroplast accumulation in the U region of Lt petals accounted for the green pigmentation in this zone (Fig. 2a; Supplementary Fig. S5). In Lt (M region) and JZ1/2 (U and M regions) petals, the accumulation of globular orange-yellow chromoplasts in the mesophyll cells contributed to the orange‒yellow coloration patterns. Chloroplast autofluorescence was detected, with Lc petals showing overall higher fluorescence intensity than Lt and JZ1/2 petals (Fig. 2b & c). The fluorescence intensity of Lt petals was intermediate, with the U region displaying the highest signal (Fig. 2b & c). A small number of chloroplasts were also observed in various regions of the JZ1/2 petals, as indicated by the florescence signal (Fig. 2b & c). These findings suggest that the petal coloration patterns in *Liriodendron* are determined by the relative accumulation of chloroplasts and orange-yellow chromoplasts.

### Transcriptional landscape underlying petal coloration patterns in *Liriodendron*

**Figure 3:**
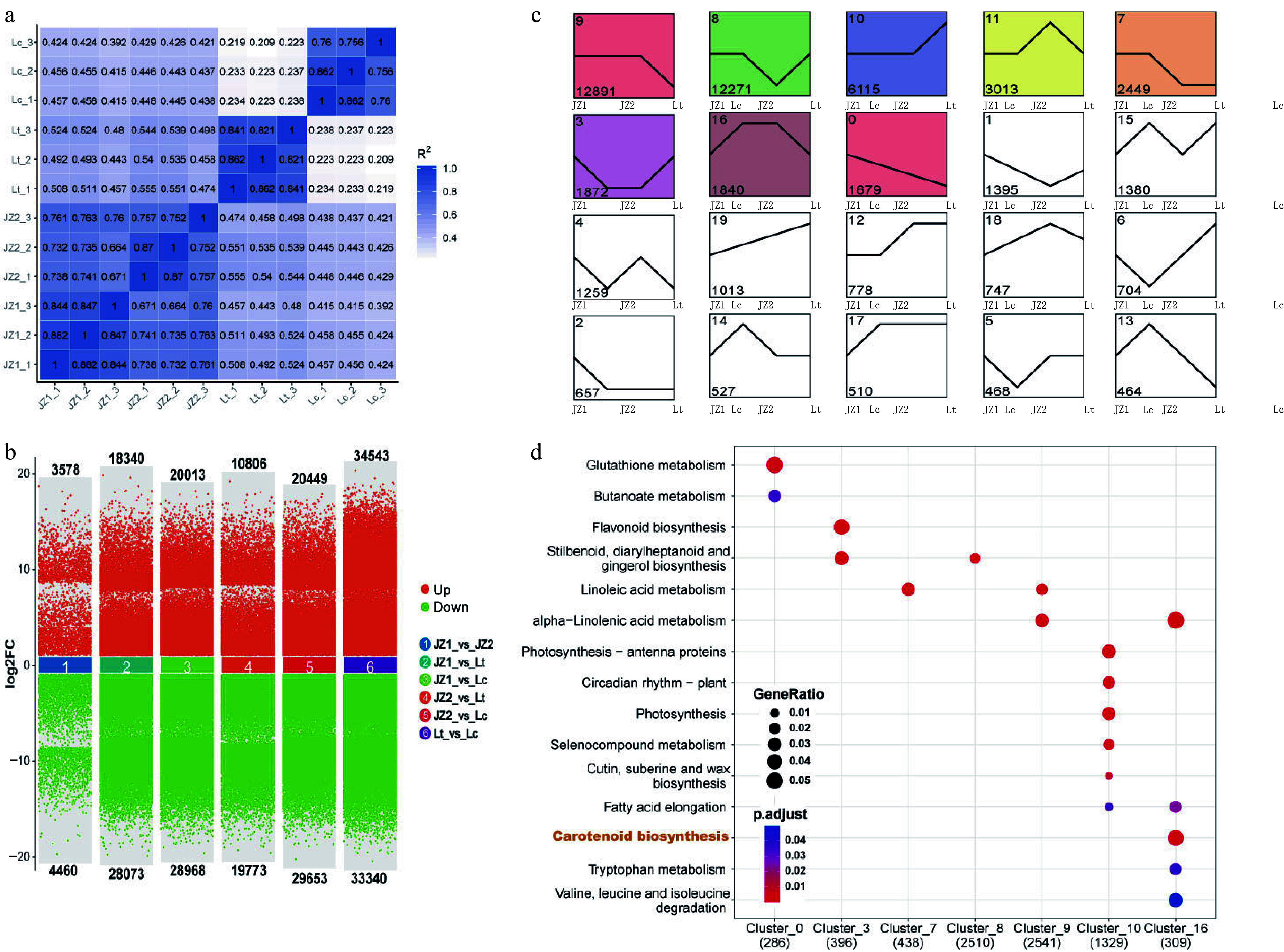
Transcriptomic analysis of *Liriodendron* petals. (a) Correlation analysis among different samples. (b) Comparative analysis of differentially expressed unigenes across multiple group comparisons. (c) Distribution of *K*-means clustering patterns of differentially expressed unigenes. Clusters are ordered according to the number of transcripts they contain. (d) KEGG enrichment analysis of unigene clusters. Dot sizes represent the proportion of unigenes within each pathway, and adjusted *p* values were calculated using the hypergeometric test.

RNA sequencing (RNA-Seq) was employed to analyze transcriptional changes in *Liriodendron* petals at the same developmental stage used for phenotypic and coloration pattern assessments. A total of 660,890,222 clean reads (99.13 Gb) were generated, with an average GC content of 48.18% (Supplementary Table S2). Since the comparative transcriptome involved different *Liriodendron* species, a *de novo* assembly strategy was adopted. Using Corset hierarchical clustering, 233,909 unigenes—represented by their longest transcript—were assembled. The N50 and N90 lengths were 1,759 bp and 496 bp, respectively (Supplementary Tables S3 & 4). All unigenes (100%) were functionally annotated using seven public databases (Supplementary Fig. S6). Sample correlation analysis showed strong intragroup consistency (*R* > 0.75) and moderate intergroup correlation between JZ1 and JZ2 samples (*R* > 0.66) (Fig. 3a). The number of differentially expressed unigenes (DEUs) varied substantially among comparison groups. Notably, Group 6 exhibited the largest number of both upregulated (34,543) and downregulated (33,340) unigenes (Fig. 3b).

To further investigate the transcriptional differences among *Liriodendron* petals, eight distinct differential expression profiles (color-coded modules) were identified through trend expression analysis (JZ1-JZ2-Lt-Lc). KEGG enrichment analysis revealed that photosynthesis-related pathways were significantly enriched in Cluster_10 (Fig. 3c), which was directly associated with chloroplast accumulation in *L. chinense*. Previous studies have shown that carotenoids play a key role in the petal coloration of *L. tulipifera*. Interestingly, the term 'carotenoid biosynthesis' was significantly enriched in Cluster_16 (Fig. 3c; Supplementary Table S5), suggesting that certain unigenes encoding carotenoid synthase-related enzymes were upregulated in JZ2 and Lt petals. To further explore the underlying cause of this discrepancy, all enrichment results across the eight expression profiles were examined. Notably, the 'carotenoid biosynthesis' term was enriched across all significantly represented clusters, with Cluster_8 and Cluster_9 containing the highest number of relevant unigenes. This suggests that these two expression patterns may play key roles in regulating carotenoid biosynthesis. Additionally, a KEGG term related to chlorophyll metabolism, 'porphyrin metabolism' —was enriched in all clusters, particularly in Cluster_8 and Cluster_9. These findings indicate that the variation in *Liriodendron* petal coloration patterns may result not only from carotenoid biosynthesis but also from the synthesis and degradation of chlorophyll (Fig. 3d).

### Key enzymes involved in carotenoid biosynthesis during petal coloration

To identify the key carotenoid biosynthetic enzymes contributing to petal coloration pattern variation in *Liriodendron,* unigenes involved in carotenoid biosynthesis and degradation were examined across different samples. Geranylgeranyl pyrophosphate (GGPP), synthesized via the plant MEP (2-C-methyl-D-erythritol 4-phosphate) pathway, serves as the direct precursor for carotenoid biosynthesis. Unigenes associated with the conversion of D-glyceraldehyde 3-phosphate (G3P) to dimethylallyl diphosphate (DMAPP), including *DXR_1/3*, *DXS_4/5/11*, *ISPD_1/2*, *ISPF*, *GCPE_2,* and *ISPH* (Supplementary Table S6), exhibited diverse expression patterns in *Liriodendron* petals. Among these, GGPS_5 (geranylgeranyl diphosphate synthase), which catalyzes the formation of GGPP, was highly expressed in all samples, indicating sufficient substrate availability for carotenoid biosynthesis (Supplementary Table S6).

**Figure 4:**
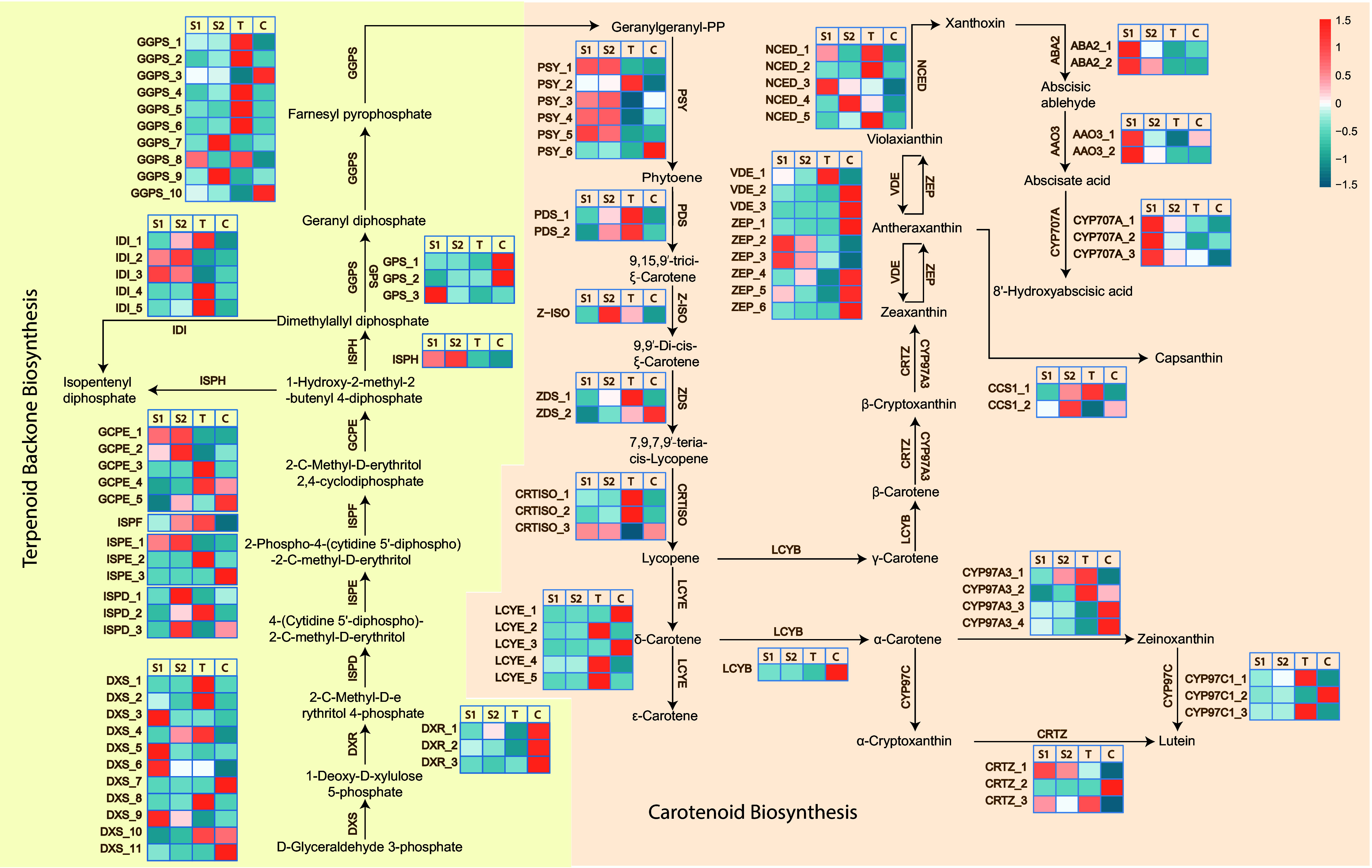
Carotenoid and terpenoid backbone biosynthesis pathways and expression profiles of related differentially expressed unigenes in *Liriodendron* petals. Heatmap shows relative expression profiles (blue-white-orange) of carotenoid biosynthesis-related unigenes in *Liriodendron* petals. DXS, 1-deoxy-D-xylulose-5-phosphate synthase; DXR, 1-deoxy-D-xylulose-5-phosphate reductor isomerase; ISPD, 2-C-methyl-D-erythritol 4-phosphate cytidylyltransferase; ISPE, 4-(cytidine 5′-diphospho)-2-C-methyl-D-erythritol kinase; ISPF, 2-C-methyl-D -erythritol 2,4-cyclodiphosphate synthase; GCPE, (E)-4-hydroxy-3-methylbut-2-enyl diphosphate synthase; ISPH, (E)-4-hydroxy-3-methylbut-2-enyl diphosphate reductase; IDI, isopentenyl diphosphate; GGPS, geranylgeranyl diphosphate synthase; GPS, geranyl diphosphate synthase; PSY, phytoene synthase; PDS, phytoene desaturase; ZDS, *ζ*-carotene desaturase; CRTISO, carotenoid isomerase; LCYE, lycopene *ε*-cyclase; LCYB, lycopene *β*-cyclase; CRTZ, *β*-carotene hydroxylase; CYP97C1, carotene *ε*-monooxygenase; CYP97A3, *β*-ring hydroxylase; ZEP, zeaxanthin epoxidase; VDE, violaxanthin de-epoxidase; NCED, 9-cis-epoxycarotenoid dioxygenase. ABA2, xanthoxin dehydrogenase; AAO3, abscisic-aldehyde oxidase; CYP707A, (+)-abscisic acid 8'-hydroxylase; CCS1, capsanthin synthase. S1, S2, T, and C represent JZ1, JZ2, Lt, and Lc, respectively. The pathway diagram was adapted from KEGG (www.kegg.jp).

The conversion of GGPP to lycopene involves multiple enzymatic steps, including catalytic synthesis by PSY, isomerization by Z-ISO and CRTISO, and desaturation by PDS, ultimately leading to lycopene biosynthesis in plastids. LCYE (lycopene *ε*-cyclase) and LCYB (lycopene *β*-cyclase) are the key rate-limiting enzymes responsible for converting lycopene into *α-*, *β*-, and *ε*-carotene. The overall low expression of *LCYB* in *Liriodendron* petals suggests that *LCYB* may not contribute significantly to *β*-carotene synthesis in *L. tulipifera* or *L. sino-americanum* (Supplementary Table S7). In contrast, *LCYE* exhibited more distinct species-specific expression patterns: *LCYE_1* and *LCYE_3* were upregulated in *L. chinense* petals, while *LCYE_2*, *LCYE_4*, and *LCYE_5* showed relatively high expression levels in Lt petals. Among them, *LCYE_4* was also expressed in JZ1 and JZ2 petals (Fig. 4; Supplementary Table S7). These expression patterns may influence the accumulation of various carotene types.

In the lutein biosynthesis pathway, lycopene is first converted to *α-*carotene by LCYE and LCYB. Subsequently, *α-*carotene is transformed into zeinoxanthin and *α*-cryptoxanthin by CYP97A (cytochrome P450-type monooxygenase 97A) and CYP97C, respectively. Finally, *CYP97C* and CRTZ (*β*-carotene 3-hydroxylase) further catalyze the conversion of zeinoxanthin and *α*-cryptoxanthin into lutein. Interestingly, *CRTZ_2* was highly expressed in Lc petals, whereas *CRTZ_3* showed high expression levels in Lt and JZ1/2 petals. Previous studies have indicated that the accumulation of zeaxanthin is one of the main factors contributing to the yellow coloration of tomato pulp. Therefore, the elevated expression of *CRTZ_3* may promote the conversion of *β*-carotene to zeaxanthin, thereby influencing the pigmentation of JZ1/2 and Lt petals. Additionally, nine unigenes encoding ZEP (zeaxanthin epoxidase) and VDE (violaxanthin de-epoxidase) were identified, among which, *ZEP_6* and *VDE_2* exhibited relatively high expression levels in Lc petals. This differential expression may affect zeaxanthin homeostasis during the xanthophyll cycle. Collectively, the variation in carotenoid synthase gene expression in *Liriodendron* petals appears to be driven primarily by two factors: species-specific expression of *LCYE* unigenes and divergent expression patterns among *CRTZ* unigenes. Both gene groups likely play critical roles in determining petal coloration.

### Key enzymes involved in chlorophyll biosynthesis and degradation during petal coloration

**Figure 5:**
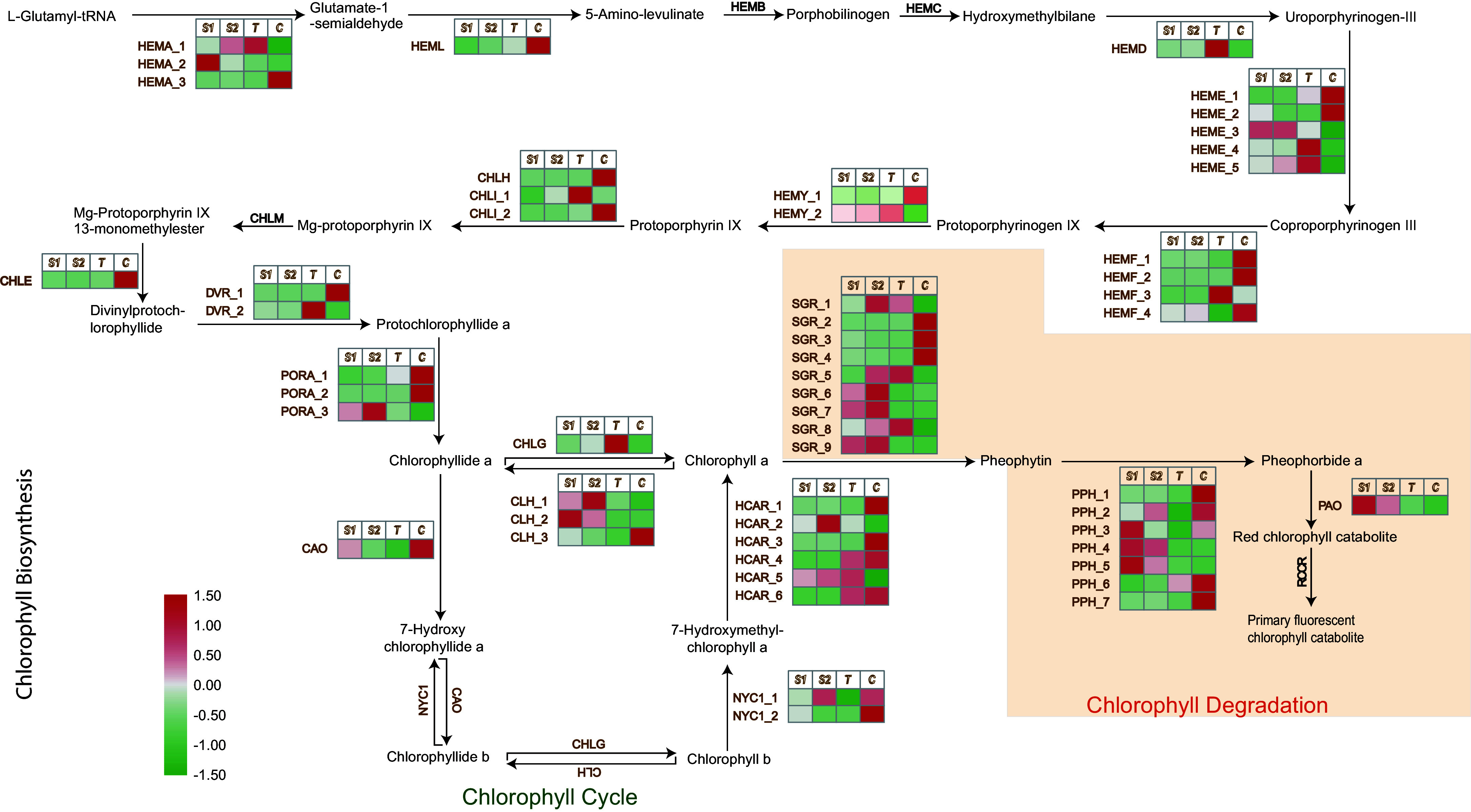
Chlorophyll biosynthesis and degradation pathways and expression profiles of related differentially expressed unigenes in *Liriodendron* petals. Heatmap shows relative expression profiles (green-white-red) of chlorophyll biosynthesis and degradation pathway unigenes in *Liriodendron* petals. HEMA, glutamyl-tRNA reductase 1; HEML, glutamate-1-semialdehyde 2,1-aminomutase; HEMB, delta-aminolaevulinic acid dehydratase 1; HEMC, porphobilinogen deaminase; HEMD, uroporphyrinogen-III synthase; HEME, uroporphyrinogen decarboxylase 1; HEMF, coproporphyrinogen III oxidase; HEMY, geranylgeranyl diphosphate synthase; CHLH, magnesium-chelatase subunit H; CHLI, magnesium-chelatase subunit I; CHLM, magnesium-protoporphyrin O-methyltransferase; CHLE, anaerobic magnesium-protoporphyrin IX monomethyl ester cyclase; DVR, divinyl chlorophyllide a 8-vinyl-reductase; PORA, protochlorophyllide reductase A; CAO, protochlorophyllide reductase A; NYC1, chlorophyll (ide) b reductase; CHLG, chlorophyll synthase; CLH, chlorophyllase; HCAR, 7-hydroxymethyl chlorophyll a reductase; SGR, STAY-GREEN; PPH, pheophytins. PAO, pheophorbide a oxygenase; RCCR, red chlorophyll catabolite reductase. S1, S2, T, and C represent JZ1, JZ2, Lt and Lc, respectively. The pathway diagram was adapted from KEGG (www.kegg.jp).

The key enzymes responsible for chlorophyll biosynthesis—from the initial substrate L-glutamyl-tRNA to the formation of chlorophyll *a* and *b* in higher plants—have been well characterized, particularly in model species such as *A. thaliana*. In *Liriodendron* petals, multiple unigenes involved in these steps were identified. The conversion of L-glutamyl-tRNA to protoporphyrin IX requires a series of enzymatic reactions catalyzed by enzymes including HEMA (glutamyl-tRNA reductase), HEML (glutamate-1-semialdehyde 2,1-aminomutase), HEMB (porphobilinogen synthase), HEMC (hydroxymethylbilane synthase), HEMD (uroporphyrinogen-III synthase), HEME (uroporphyrinogen decarboxylase), HEMF (coproporphyrinogen III oxidase), and HEMY (protoporphyrinogen/coproporphyrinogen III oxidase) (Fig. 5).

Among these enzymes, the catalytic enzymes HEMY_1 and HEMY_2, which mediate the final step of protoporphyrin IX (Proto IX) synthesis (Supplementary Table S8), were highly expressed in the petals. MgCH (magnesium chelatase), composed of three subunits—CHLH (magnesium chelatase subunit H), CHLI (magnesium chelatase subunit I), and CHLD (magnesium chelatase subunit D)—subsequently catalyzes the insertion of magnesium into Proto IX, generating Mg-protoporphyrin IX (MgP IX) (Fig. 5). *CHLH* expression was notably higher in Lc petals compared to the others, facilitating the catalytic conversion of Proto IX. MgP IX is further converted to protochlorophyllide (Pchlide) by CHLM (magnesium-protoporphyrin O-methyltransferase), CHLE (magnesium-protoporphyrin IX monomethyl ester (oxidative) cyclase), and *DVR* (divinyl chlorophyllide 8-vinyl-reductase). Both *CHLE* and *DVR_1* showed higher expression in Lc petals relative to the other samples. Subsequently, three unigenes encoding POR (protochlorophyllide reductase) were identified; among them, *PORA_1* and *PORA_2* were strongly upregulated in Lc petals, catalyzing the reduction of Pchlide a to chlorophyllide (Chlide) *a*. Finally, Chlide *a* is either directly converted to chlorophyll (Chl) *a* by CHLG (chlorophyll synthase) or oxidized to Chlide *b* by CAO, followed by conversion to Chl *b* by CHLG. These findings indicate that *PORA_1* and *PORA_2* act as key reductases limiting chlorophyll biosynthesis in the orange-yellow regions of *Liriodendron* petals*.*

In addition, the varying expression levels of chlorophyll-degrading unigenes influence chlorophyll content. In this process, nine and seven differentially expressed unigenes encoding SGR (magnesium dechelatase) and PPH (pheophytinase), respectively, were identified (Fig. 5; Supplementary Table S8). *SGR_7* and *SGR_8* were upregulated in the petals of Lt and JZ1/2, promoting the conversion of Chl *a* to pheophytin. Furthermore, the expression pattern of *PPH_2*, which catalyzes the conversion of pheophytin to pheophorbide a, corresponded well with the petal coloration gradient: JZ1/2 > Lt > Lc. Pheophorbide a was subsequently oxidized to a red chlorophyll catabolite through the differential expression of PAO (pheophorbide a oxygenase) in *Liriodendron* petals.

### Metabolism of carotenoids and chlorophyll during petal coloration

**Figure 6:**
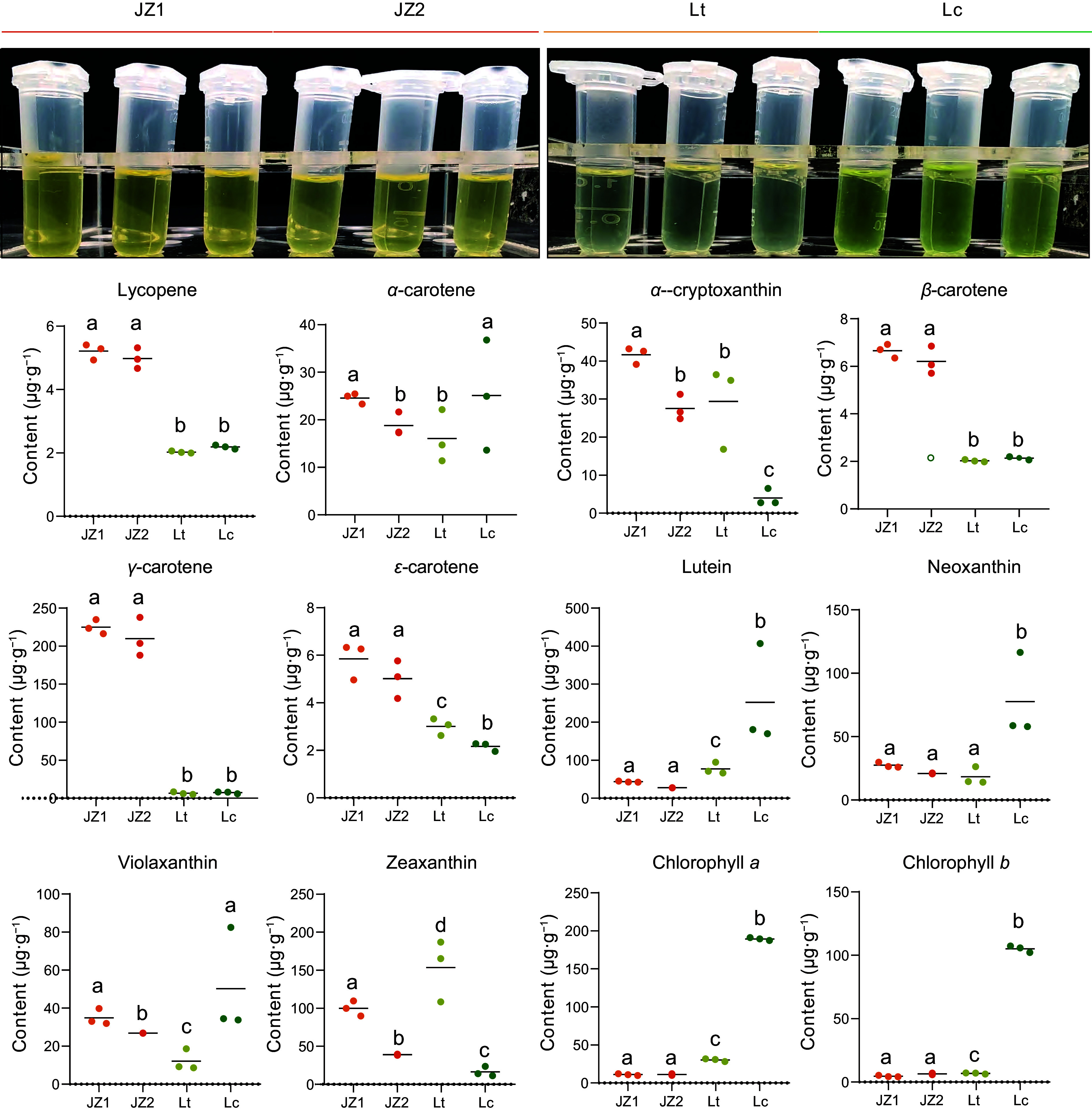
Statistical analysis of carotenoid and chlorophyll contents in *Liriodendron* petals. Data were analyzed using two-way ANOVA followed by Tukey's post-hoc test. Results are presented as mean ± SD. Different letters indicate statistically significant differences (*p* < 0.05).

To further confirm the roles of carotenoids and chlorophyll in *Liriodendron* petal coloration, the metabolic contents of 13 compounds were measured in the same samples used for the transcriptome analysis (Supplementary Table S9). The level of lycopene, a key substrate for downstream carotenoid biosynthesis, was higher in JZ1/2 petals compared to the other petals and remained stable in Lc and Lt petals (Fig. 6).

Moreover, *ε*-, *β*-, and *γ*-carotene were also upregulated in JZ1/2 petals, with *γ*-carotene showing an exceptionally high content in JZ1/2 petals. Meanwhile, no significant difference in *γ*-carotene accumulation was detected between Lt and Lc petals. Additionally, the abundance patterns of five xanthophylls (lutein, *α*-zeinoxanthin, neoxanthin, violaxanthin, and zeaxanthin) varied among samples. Compared with Lt and JZ1/2 petals, lutein and neoxanthin contents were higher in Lc petals. *α*-Zeinoxanthin, a derivative of *α*-carotene and precursor to lutein, exhibited low accumulation in Lc. Among chlorophyll metabolites, the contents of both Chl *a* and Chl *b* were significantly increased in Lc petals, with Chl *a* content being higher in Lt petals than in JZ1/2 (Fig. 6). In summary, these results suggest that the orange-yellow region of the petals results from carotenoid accumulation in the chromoplast of petal parenchyma cells, whereas chlorophyll biosynthesis and accumulation in the chloroplasts underlie the dark green coloration of the petals, which is also accompanied by carotenoid biosynthesis on the thylakoid membranes.

### Global transcriptional patterns of key enzymes during color changes in *Liriodendron* petals

**Figure 7:**
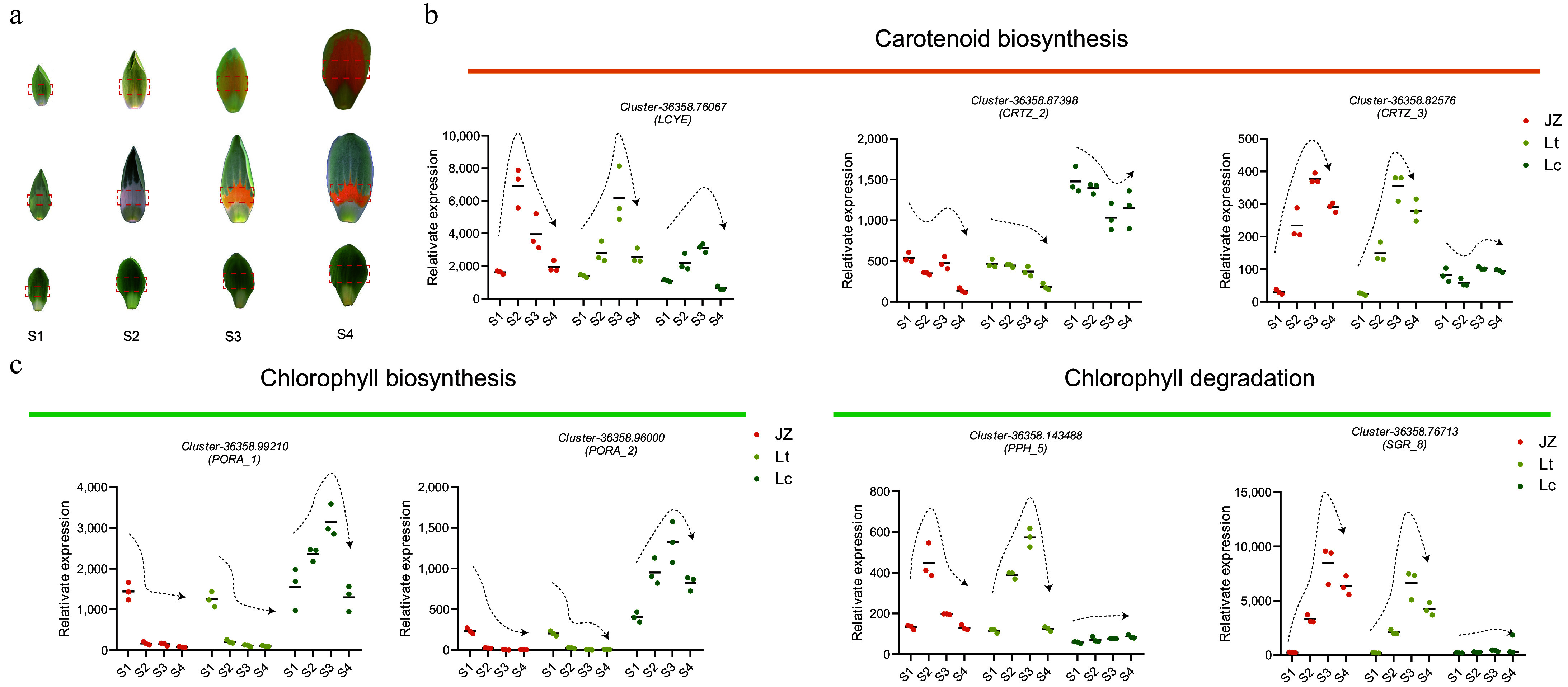
Dynamic expression patterns of key carotenoid and chlorophyll biosynthesis unigenes in the central petal region during *Liriodendron* flower development. (a) Coloration patterns of the central petal region across development stages (S1−S4) of *Liriodendron* flowers. (b), (c) Temporal expression profiles of target unigenes in the central petal region.

Previous studies have shown that petal coloration-related genes in *L. tulipifera* are initially upregulated and subsequently downregulated during flower development. Therefore, candidate unigenes associated with carotenoid and chlorophyll metabolism require further validation to account for interspecies variation. To validate the transcriptome results, qRT‒PCR was conducted using tissues from the pigmented central regions of the petals at various developmental stages. Given the similarity in petal coloration patterns among the samples, only the JZ1 cultivar was selected for validation. The results confirmed that the expression of *Z-ISO* and *ZDS_1* peaked and then declined during flower development in all examined *Liriodendron* species (Fig. 7)*.*

Compared with other petals, *CRTISO* expression began to increase at the S2 stage in JZ1 and Lt petals (Supplementary Fig. S7), while remaining consistently low in Lc petals. These findings suggest that lycopene accumulates as a substrate for carotenoid biosynthesis in petals. The subsequent cyclization of lycopene into downstream carotenes is catalyzed by the rate-limiting enzymes LCYB and LCYE (Fig. 7). Among these, *LCYB* exhibited stable expression across developmental stages, indicating that it does not contribute to the differential accumulation of carotenes (Supplementary Fig. S7). In contrast, *LCYE* displayed species-specific expression patterns across multiple transcripts. Sequence analysis revealed high conservation among *LCYE* unigenes (Supplementary Fig. S1 & S2). Interestingly, *LCYE* displayed transient upregulation followed by downregulation during flower development, with significantly lower expression in Lc petals compared to Lt and JZ1 at the corresponding stages (Fig. 7). Additionally, *CRTZ_2* and *CRTZ_3* exhibited opposing expression patterns: *CRTZ_2* maintained relatively high expression in Lc petals, while *CRTZ_3* expression increased initially and then declined during flower development in JZ1 and Lt petals. In the chlorophyll biosynthesis pathway, the key PORA enzymes, which convert Pchlide *a* to Chlide *a*, were repressed during the formation of the orange-yellow region of the petals. Conversely, the chlorophyll degradation-related genes *SGR_8* and *PPH_5* were strongly upregulated during the color change in Lt and JZ1 petals but remained stably expressed in Lc. Collectively, these dynamic expression analyses indicate that petal coloration in *Liriodendron* is coordinately regulated by both chlorophyll and carotenoid metabolism.

### Silencing of *LcPORA* genes impairs chlorophyll biosynthesis and chloroplast development in *Liriodendron* petals

**Figure 8:**
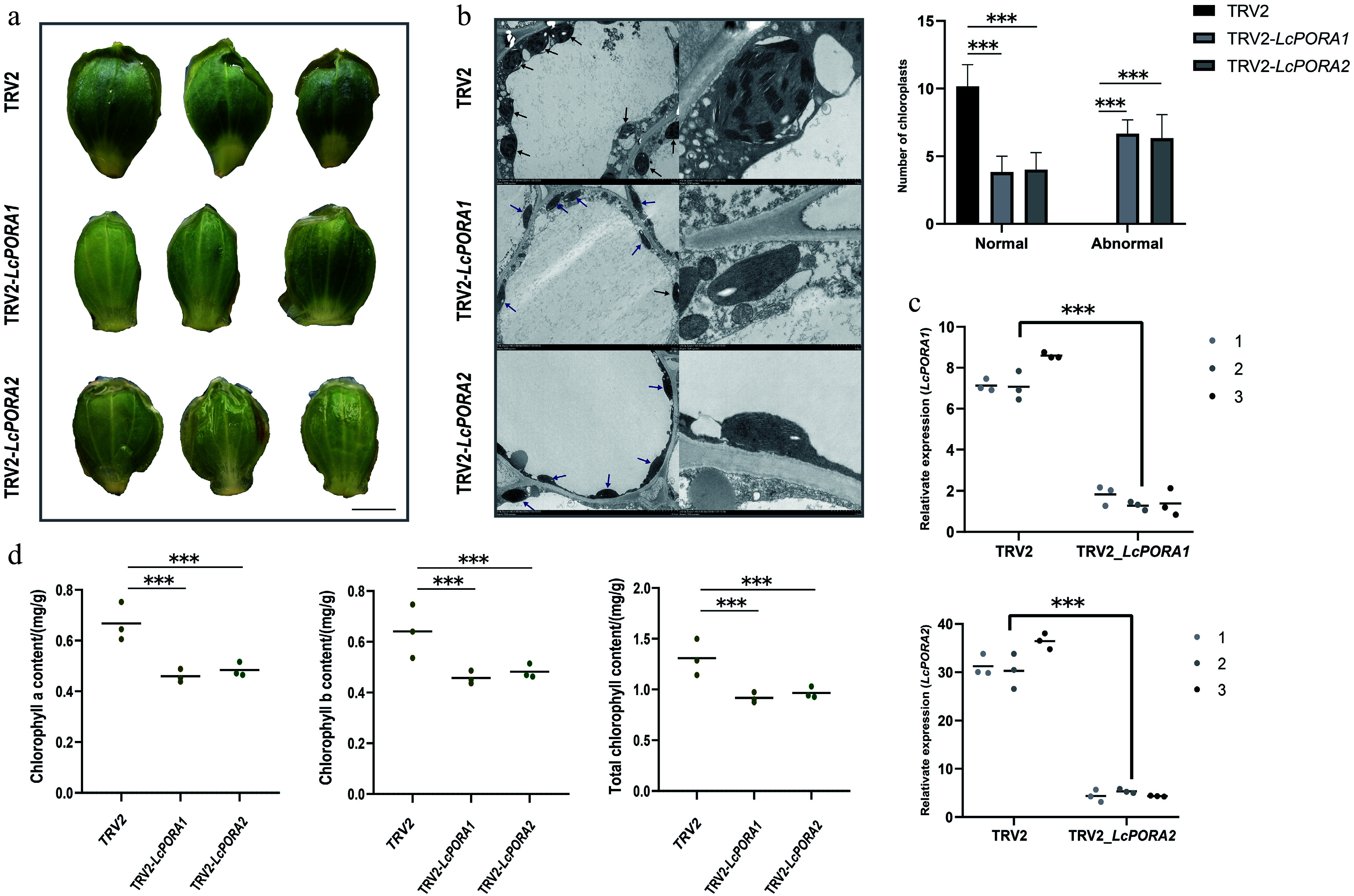
Phenotypic alterations induced by silencing *LcPORA1* and *LcPORA2* in *L. chinense* petals. (a)−(c) Downregulation of *LcPORA1* and *LcPORA2* expression via VIGS technology significantly affected petal coloration and chloroplast structure in *L. chinense.* Black arrows indicate normal chloroplasts, while blue arrows indicate abnormal chloroplasts. Scale bar = 1 cm. (d) Downregulation of *LcPORA1* and *LcPORA2* significantly reduced the chlorophyll content in *L. chinense* petals*.* Data were analyzed using two-way ANOVA followed by Tukey's post-hoc test. Results are presented as mean ± SD. *** indicates *p* < 0.001.

Transcriptional dynamics revealed that *LcPORA_1* and *PORA_2* expression in non-*L. chinense* petals drastically declined to minimal levels at the S2 stage, suggesting that inhibition of chlorophyll biosynthesis is a critical contributor to petal color transitions in *Liriodendron*. To test this hypothesis, a virus-induced gene silencing (VIGS) system was established for the first time using isolated *Liriodendron* petals. Silencing either *LcPORA_1* or *LcPORA_2* in *L. chinense* significantly impaired the petal color transition, leading to a shift from dark-green to light-green pigmentation (Fig. 8a & c).

In *L. chinense* petals, chloroplasts were oval-shaped with neatly stacked thylakoids. However, silencing either *LcPORA_1* or *LcPORA_2* led to a reduced number of chloroplasts per cell, and the chloroplasts became fusiform and significantly smaller than those in normal petals. Ultrastructural analysis revealed disorganized thylakoids and blurred grana-stroma differentiation (Fig. 8b). Moreover, chlorophyll content was significantly decreased (Fig. 8d). These results demonstrate that downregulation of either *LcPORA_1* or *LcPORA_2* inhibits chlorophyll biosynthesis and disrupts chloroplast ultrastructure in *Liriodendron* petals.

## Discussion

### Chlorophyll biosynthesis and degradation initiate petal coloration changes in *Liriodendron*

Phenotypic observations revealed distinct pigmentation dynamics among *Liriodendron* petals. In *L. tulipifera*, color transitioned in a staged manner within well-defined bands (Fig. 7a). Interestingly, band formation occurred before visible pigment deposition, suggesting a developmental pre-pattern rather than pigment-derived banding. In contrast, JZ1 petals lacked banding, exhibiting a gradual transition from light green to uniform orange-yellow, driven by marginal expansion and chlorophyll degradation. Microscopic and fluorescence analyses showed that green regions contained abundant chloroplasts, whereas orange-yellow areas accumulated chromoplasts with reduced chloroplast autofluorescence (Fig. 2). The morphological diversity of plastids in different color regions suggests a tightly regulated chloroplast-to-chromoplast conversion mechanism.

Integrated transcriptomic and metabolomics analyses revealed that persistent chlorophyll accumulation maintains green coloration, while carotenoid deposition in chromoplasts drives the development of orange-yellow bands. Although carotenoids were also deposited in chloroplasts within green regions, they likely function in light harvesting and chlorophyll protection, rather than as direct contributors to visible coloration, which is similar to findings in *Momordica charantia* seedlings^[32]^. The sharp downregulation of *PORA_1* and *PORA_2* coincided with the onset of color transition (Fig. 7), suggesting that inhibition of chlorophyll synthesis initiates petal depigmentation. This decrease in *PORA_1* and *PORA_2* expression was associated with a reduction in chloroplast number and altered ultrastructure (Fig. 8), suggesting that the *PORA* genes help stabilize chloroplast structure, and that their downregulation may trigger chloroplast degradation. Additionally, the chlorophyll degradation genes *SGR_8* and *PPH_5* were strongly upregulated after stage S1. SGR is known to destabilize the chlorophyll-apolipoprotein complex of PSII during chloroplast senescence by interacting with catabolic enzymes^[33,34]^, and it also modulates lycopene and *β*-carotene levels through direct interaction with SlPSY1 in tomato^[35]^. PPH hydrolyses pheophytin and promotes light-harvesting complex breakdown in the PAO pathway^[36,37]^. Together, these findings suggest that inhibited chlorophyll biosynthesis combined with enhanced degradation promotes chloroplast disassembly during petal development, ultimately leading to the observed changes in *Liriodendron* petal coloration.

### Carotenoid accumulation in chromoplasts drives color changes in *Liriodendron* petals

Carotenoids are lipophilic pigments stored in plastids, particularly chromoplasts, where they contribute to red, orange, and yellow coloration in plant tissues^[6]^. Variations in carotenoid type and content among plant tissues lead to significant differences in chromoplast morphology and coloration^[38]^. Chromoplasts vary in shape—spherical, tubular, membranous, or crystalline, depending on the type and content of carotenoids. For instance, lycopene and phytoene in ripened tomato fruits form globular structures, while *β*-carotene and zeaxanthin accumulate in rod-shaped membranes^[39]^. In broccoli, mutation of the *ORANGE* gene leads to *β*-carotene buildup and orange pigmentation, accompanied by flake-like plastid structures^[40]^. Similarly, high violaxanthin levels in chili peppers lead to the formation of fusiform chromoplasts^[41]^. Intriguingly, different *β*-carotene isomers alter plastid morphology, with cis-trans isomers tending to accumulate in spherical and crystalline chromoplasts^[42]^.

During carotenoid biosynthesis in *Liriondedron* petals, the upregulation of enzymes in the lycopene biosynthesis pathway ensures a sufficient substrate pool for downstream carotenoids. Compared to the relatively stable expression of *LCYB* throughout petal development, *LCYE* expression exhibited dynamic and diverse patterns. The differential accumulation of *α*-, *β*-, and *ε*-carotene in petals suggested that LCYE may catalyze lycopene into various carotenoid branches, a function also observed in carrot roots^[43]^. Notably, *γ*-carotene-a key intermediate in the lycopene-to-*β*-carotene conversion-accumulated excessively in JZ1/2 petals. This likely resulted from a metabolic imbalance in late-stage petal development, where the lycopene biosynthesis rate exceeded its conversion rate. The accumulation of excess lycopene may have caused substrate inhibition of LCYE activity, leading to abnormal accumulation of metabolic intermediates^[44]^. Additionally, multiple xanthophylls exhibited differential accumulation patterns in JZ1/2 petals. These findings indicate that the appearance of orange-yellow pigmentation in *Liriodendron* petals is not attributed to a single pigment but instead arises from the coordinated and differential accumulation of multiple carotenoids within the chromoplast.

### Understanding the molecular mechanisms underlying petal color change in *Liriodendron* facilitates the genetic improvement of ornamental varieties

To adapt to complex environmental changes, angiosperms have evolved diverse petal traits-including shape, color, and number—over time^[45,46]^. In *Liriodendron*, *L. sino-americanum* exhibits novel flower types and pigmentation patterns, with petals predominantly covered by orange-yellow hues. Compared to *L. tulipifera* and *L. sino-americanum*, *L. chinense* shows markedly smaller petals, both in size and pigmented area. The temporal stability of petal hue in *L. chinense*, alongside the dynamic, spatial-temporal pigmentation patterns observed in *L. tulipifera*, offers a valuable framework for understanding gene recombination and regulation during the hybrid flower development across time and tissue domains.

This work provides a molecular and developmental basis for future breeding efforts aimed at enhancing the ornamental values of *Liriodendron*. Furthermore, petal color plays a vital ecological role by attracting pollinators to ensure reproduction success^[1]^. The diversification of petal coloration in *Liriodendron* may also reflect adaptations to habitat shifts following lineage divergence in the middle to late Miocene. Population resequencing indicates that *L. chinense* became geographically restricted to mountainous regions of East Asia after repeated glaciations, leading to long-term isolation and population decline^[47]^. In contrast, the population size of *L. tulipifera* rebounded after the Quaternary glaciation, aided by the more open terrain of eastern North America, which allowed for greater connectivity and gene flow^[25]^. This broader ecological niche may have driven the evolution of specialized floral traits in *L. tulipifera*, promoting adaptation and reproduction success-also reflected in the distinct nectar secretion patterns of *L. chinense* and *L. tulipifera*^[48]^*.*

## Conclusions

**Figure 9:**
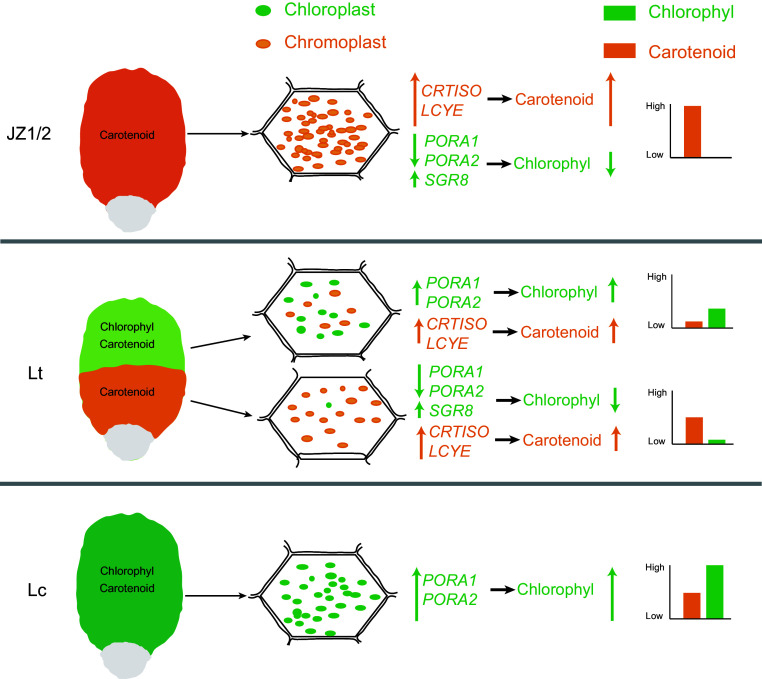
Schematic illustration of petal color variation in *Liriodendron*. The green and orange elliptical spheres represent chloroplasts and chromoplasts, respectively. The green and orange on the coordinate axis represent the relative content of chlorophyll and carotenoids, respectively.

This study demonstrates that the formation of orange-yellow bands in *Liriodendron* petals is primarily driven by dynamic plastid transformation involving chloroplast degradation and chromoplast biogenesis. Downregulation of *PORA_1* and *PORA_2* disrupts chloroplast structure and chlorophyll biosynthesis, while altered expression of *SGR_8* and *PPH_5* accelerates chlorophyll degradation. Concurrently, the upregulation of carotenoid biosynthetic genes, particularly *CRTISO* and *LCYE*, promotes carotenoid accumulation in developing chromoplasts, leading to visible pigmentation (Fig. 9). These findings reveal the key molecular regulators of petal coloration in *Liriodendron* and provide valuable targets for the genetic improvement of ornamental traits.

## SUPPLEMENTARY DATA

Supplementary data to this article can be found online.

## Data Availability

The datasets generated during and/or analyzed during the current study are available from the corresponding author on reasonable request.
