# Supplementary material for: PORA1/2-dependent chlorophyll biosynthesis coordinates with carotenoid accumulation to drive petal color patterning in Liriodendron
Source: For Res (Fayettev). 2025 Jul 14;5:e013. doi: 10.48130/forres-0025-0013 (PMC12441795; doi:10.48130/forres-0025-0013)
Supplement: Supplementary file 1 — Supplementary data to this article can be found online. [file FR-2025-5-0013-Supplementary.zip › 10.48130_forres-0025-0013-Suppl-FigureS2.pdf]

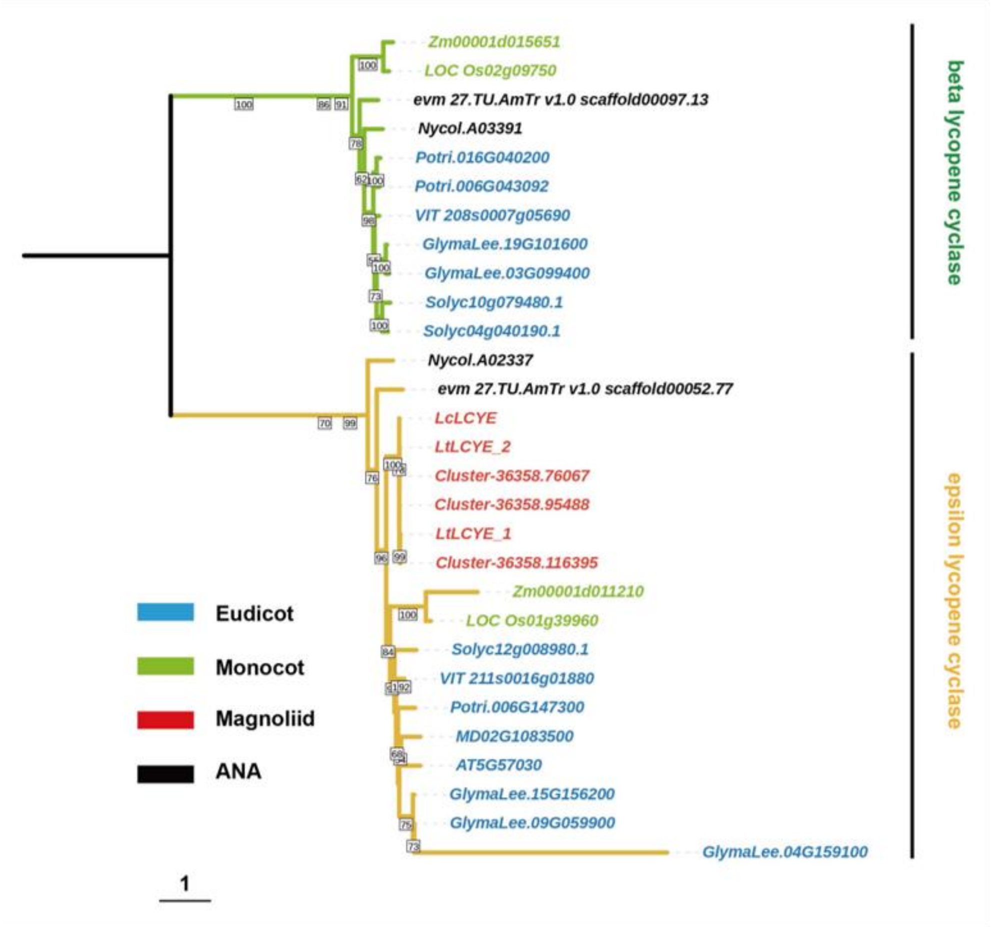

Fig. S2 Phylogenetic tree of LCY genes constructed based on 12 plant species.

The phylogenetic tree was constructed by IQtree2 using the maximum likelihood (ML) method (1,000 bootstrap), and the optimal model was Q. plant+G4.
