# Supplementary figures and images for: PORA1/2-dependent chlorophyll biosynthesis coordinates with carotenoid accumulation to drive petal color patterning in Liriodendron
Source: For Res (Fayettev). 2025 Jul 14;5:e013. doi: 10.48130/forres-0025-0013 (PMC12441795; doi:10.48130/forres-0025-0013)

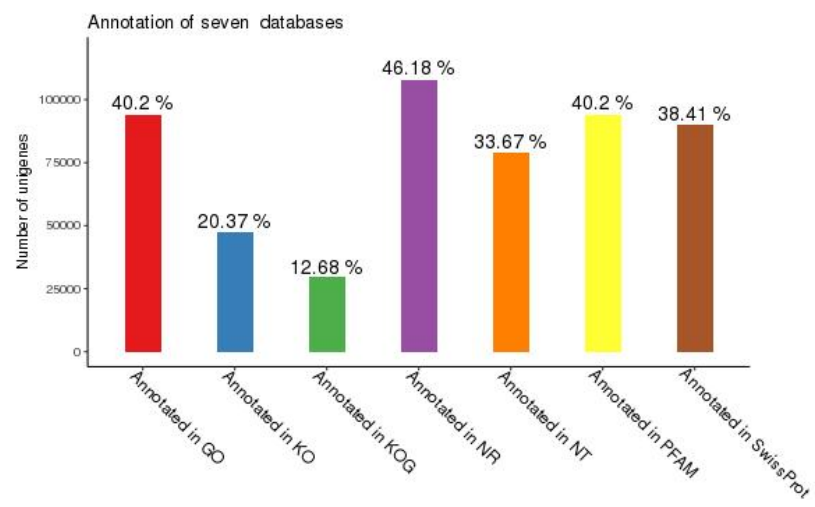

Fig. S6. Unigenes functional annotation statistics

Supplement: Supplementary file 1 — Supplementary data to this article can be found online. [file FR-2025-5-0013-Supplementary.zip › 10.48130_forres-0025-0013-Suppl-FigureS6.pdf]
